# A high-throughput approach to optimize and understand nanoparticle protein degraders

**DOI:** 10.1039/d5na00811e

**Published:** 2025-11-17

**Authors:** Joppe Oldenburg, Marrit M. E. Tholen, Janne G. D. Donkers, Ana Ortiz-Perez, Valentina Girola, Lorenzo Albertazzi

**Affiliations:** a Department of Biomedical Engineering, Institute for Complex Molecular Systems (ICMS), Eindhoven University of Technology Eindhoven 5612AZ The Netherlands l.albertazzi@tue.nl

## Abstract

Targeted protein degradation strategies have emerged as a promising approach for addressing undruggable membrane proteins by redirecting them to the cell's degradation machinery. Traditionally, this has been achieved through antibody-based constructs that hijack the endocytic pathways. More recently, nanoparticles have been proposed as a modular platform for surface protein scavenging, offering tunable properties and multivalent targeting. However, the extent to which nanoparticle physicochemical features influence degradation efficacy remains to be elucidated. In this study, we set up a high-content imaging workflow to screen a diverse library of antibody-functionalized nanoparticles varying in material composition, size, and targeting modality. Our results reveal that specific combinations of nanoparticle properties induce efficient receptor scavenging, allowing us to understand structure–activity relations better. These findings highlight the complex interplay between nanoparticle design and biological response and demonstrate the value of our high-throughput platform for guiding the rational design of nanoparticle-based protein degraders.

## Abstract 2

Targeted protein degradation strategies in the form of nanoparticles are a promising approach for addressing undruggable membrane proteins by redirecting them to the cell's degradation machinery.

## Graphical abstract

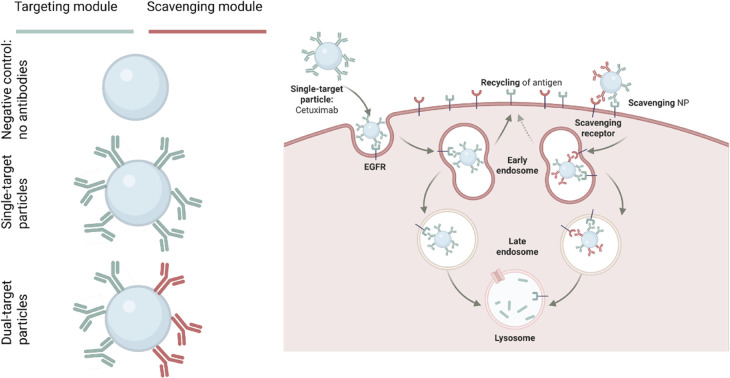

## Introduction

In recent years, selective degradation of proteins of interest *via* targeted protein degradation (TPD) has gained a lot of interest. With this approach, a target protein which is linked to disease progression is forced into the intracellular protein degradation machinery. This induced protein degradation can modulate cell signalling and function, resulting in a targeted therapeutic effect.^1,2^ The first and second generation TPDs depend on the ubiquitin-protease system (UPS), also named proteolysis chimeras (PROTACs), and are mostly designed for protein targets in the cytosol.^3^ However, targeting membrane proteins is equally relevant and new alternative approaches have been developed such as lysosome-targeting chimeras (LYTACs).^4^ These constructs consist of a protein-targeting module (*e.g.* antibody) tethered to a mannose-6-phosponate, which effectively hijacks the cell's degradation pathways to completely remove proteins from the cell membrane. All these approaches have a bifunctional modular structure in common, targeting the protein of interest on one side and interacting with the degradation machinery on the other. An example of a receptor that has been successfully ablated using LYTACs is the epidermal growth factor receptor (EGFR).^4,5^ EGFR plays a pivotal role in tumour growth, survival, and resistance mechanisms, making it a high-value target in multiple cancer types.^6^

Inspired by PROTACs and LYTACs, new modalities now aim to harness receptor trafficking and degradation for therapeutic benefit, also known as scavenging.^7^ Recent work by Liu *et al.* (2024) demonstrated that nanoparticle (NP)-based systems can be designed to mimic LYTAC functionality, using multivalent ligand presentation to trigger internalization and lysosomal trafficking of specific cell-surface receptors.^8^ These NP systems offer a flexible and scalable platform, with the potential to fine-tune receptor engagement, endocytosis rates, and downstream trafficking by adjusting particle properties. While the flexibility and modularity of nanoparticles is key for their potential as protein degraders, this also opens several questions about the design of optimal particles and how the physicochemical properties of NPs (size, shape, material, multivalency, affinity and target of the ligands) relate to biological performance.

Here, we report a high-throughput imaging approach to optimize nanoparticle protein degraders and understand their structure–activity relations. First, we generated a nanoparticle library of 130 distinct NP formulations, varying in size (50–200 nm), material (silica or polymer), ligand nature (valency and mono- or dual targeting) and valency, engineered to target and degrade the epidermal growth factor receptor (EGFR) *via* NP-mediated scavenging. Then, we screened them using a quantitative method to assess nanoparticle uptake and EGFR scavenging at the single-cell level using high-content imaging (HCI). This approach allowed us to map design–function relationships between nanoparticle features and receptor scavenging efficiency, uncovering synergistic effects between particle diameter, scavenger module identity, and anti-EGFR density. Moreover, we identified specific NP formulations that achieved significant EGFR depletion without compromising cell viability or triggering nonspecific uptake, highlighting their potential for targeted protein degradation in cancer therapy.

Overall, our study provides a high-resolution, quantitative framework for optimizing nanoparticle-mediated receptor scavenging, and underscores the value of high-content imaging in evaluating complex bio–nano interactions at scale. These findings contribute to the broader effort to develop modular, programmable systems for selective degradation of disease-relevant membrane proteins.

## Results

### Methodology

**Fig. 1:**
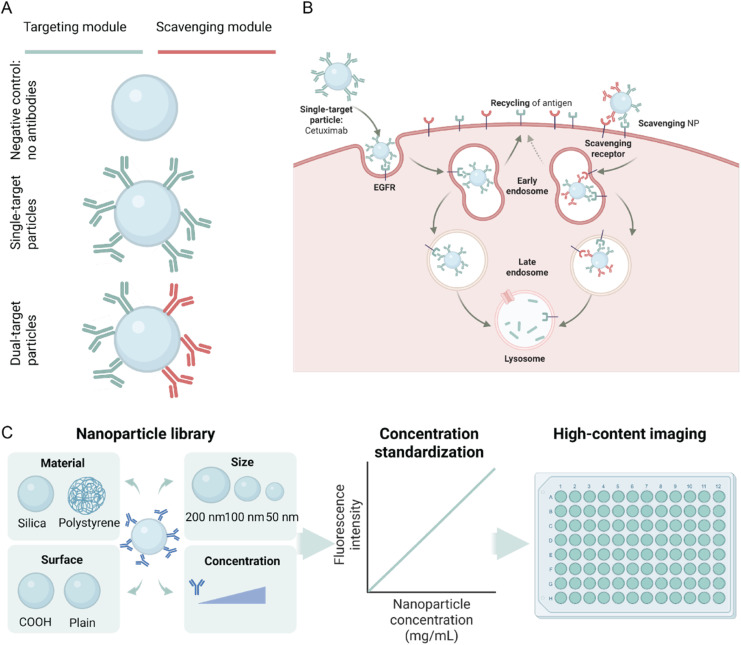
Schematic overview. (A) Nanoparticles in the library either have no antibodies, only targeting modules (single-target) or both a targeting module and a scavenging module (dual-target). Throughout this work, the targeting module is cetuximab, an antibody against EGFR. The scavenging module is either a protein or an antibody against an internalizing receptor. (B) Schematic representation of the pathway that single-target and dual-target particles are expected to follow. Single-target particles are expected to recycle the antigen back to the membrane, while dual-target particles are expected to hijack this pathway, resulting in a lowering in the expression of the receptor. (C) Overview of the workflow. First, a NP library is synthesized consisting of NPs made of different materials, with different sizes, conjugation methods and number of antibodies. Then, their concentration is normalized based on their fluorescence before they are administered to the cells. The NP uptake and the effect on EGFR expression are assessed using high-content imaging.

To demonstrate the potential of nanoparticles for scavenging of receptors, we synthesised a nanoparticle library consisting of particles with either no functionalisation, only a targeting antibody against EGFR (cetuximab (Ctx)) or both a targeting and a scavenging antibody (Fig. 1A). Fig. 1B shows the expected mechanism of action, compared between a single- and a dual-target particle. Similar to Ctx by itself, the NPs will bind to the target receptor(s) on the cell membrane, followed by cell uptake *via* endocytosis. We anticipate that the secondary scavenging antibody on the nanoparticle acts as a guide towards the lysosome, as we see with LYTAC and PROTAC methods. After a targeting antibody binds to its membrane target the complex can internalize *via* endocytosis and two pathways are possible: (i) the complex is transported to more mature endosomes and it is finally degraded in the lysosome and (ii) the membrane protein may recycle back to the membrane (with or without the antibody bound). Similarly to LYTACs we consider that dual-target nanoparticles may enhance the former and act with greater efficacy with regard to protein degradation. It has to be considered that in comparison with LYTACs the nanoparticle itself can play a role deciding between the two pathways, and may enhance degradation depending on its size and valency.

To find which features of the nanoparticles are mostly contributing to this effect, a nanoparticle library consisting of more than 130 different particles was formulated as shown in Fig. 1C. The features that were varied in this library were: (i) the material of the particle, (ii) the diameter of the particle, (iii) the conjugation method for the targeting and scavenging modules and (iv) the concentration and type of targeting and scavenging modules (Fig. 1C). Nanoparticles were either silica or a polystyrene polymer. Diameters ranged from 50 nm to 250 nm, which is a proper window in which nanoparticles are internalized.^9^ For functionalisation, two approaches were chosen: covalent attachment using EDC-conjugation or non-covalent attachment through physisorption. As a targeting moiety, cetuximab (Ctx), a therapeutic antibody against the epidermal growth factor receptor (EGFR), was used. This receptor is often overexpressed in multiple cancers and therefore is an interesting target for therapies.^6^ The concentration of this antibody was varied in 5 levels, ranging from 0.17 to 1.36 Ctx per carboxylic acid group of the NPs. Based on previous research, this roughly translates to 5 Fab domains exposed on the surface of the nanoparticle for the smaller particles, while the bigger particles can have up to roughly 50 Fab domains exposed.^10^ The corresponding concentration of antibodies was also physisorbed on the particles. Fourteen different scavenging molecules were used, targeting a variety of recycling or endocytosis-inducing receptors, which will be discussed extensively in the next sections.

For a fair comparison of nanoparticle uptake, it is important not only that the nanoparticles are fluorescently labeled, but also that the starting concentration and fluorescence are normalized.

Therefore, prior to administration of the particles to the cells, NP fluorescence and concentration were calibrated. See the SI for a list of NPs prepared, their properties and the concentration and fluorescence calibration.

Finally, both the nanoparticle uptake and the effect on EGFR expression were assessed by high-content imaging (HCI) (Fig. 1C). The automated imaging resulted in a quantitative outcome for the nanoparticle uptake and EGFR up- or downregulation.

### Proof of concept

**Fig. 2:**
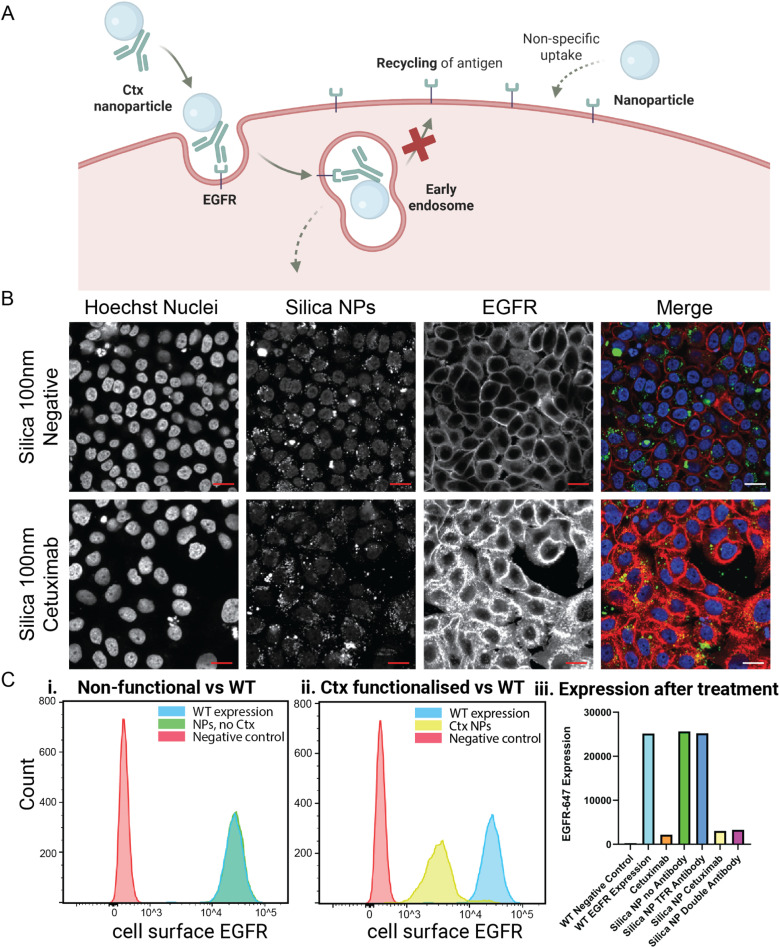
Proof of concept. (A) Schematic representation of the pathway that the single-target NPs and non-targeting particles are expected to follow, resulting in reduced EGFR expression due to scavenging of the receptor. Mode of action of the other particles is depicted in Fig. S1. (B) Representative confocal images of fluorescent 100 nm silica nanoparticles and 100 nm silica nanoparticles functionalized with cetuximab. MDA-MB-468 cells were stained with Hoechst and anti-(intracellular)EGFR. Scale bar: 20 µm. (C) Flow cytometry histograms of the EGFR expression after (i) bare silica nanoparticles (green), blue and green are overlapping, stacked histograms can be found in Fig. S2A, and (ii) Ctx functionalized NPs (yellow). The red peak depicts the negative control (no EGFR-647) and the blue peak represents WT EGFR-647 expression. (iii) Expression values of cell-membrane EGFR-647 after treatments with different types of nanoparticles (TFR antibody functionalized (dark blue) and double antibody (purple)) and Ctx alone (orange). WT: wild type expression of EGFR, Ctx: cetuximab. FACS and confocal experiments are biological replicates.

Before performing high-throughput measurements, the nanoparticles' potential to scavenge receptors was assessed through a proof of concept. For this, carboxyl-silica nanoparticles without functionalisation were compared to the same nanoparticles functionalised with Ctx (at 0.68 Ctx/COOH). It is expected that the particles without functionalisation are able to enter the cell by non-specific uptake, but they do not affect EGFR receptors. In contrast, we expect the uptake to significantly increase for particles functionalised with cetuximab. To test if this is enhanced with a scavenging modality, particles with both cetuximab and a transferrin receptor antibody are also tested, which are expected to decrease EGFR expression even further. As an extra control, particles with only TFR antibody should have no effect on EGFR expression (Fig. 2A and S1). This hypothesis was tested using both confocal microscopy (Fig. 2B) and flow cytometry (Fig. 2C). In Fig. 2B, to check for a NP effect on EGFR, cells were fixed and an anti-EGFR antibody specifically targeting EGFR through an epitope in the intracellular side (to avoid competition with the NPs) was used. One can appreciate that the treatment with Ctx-NP has a significant impact on the EGFR signal and localization. While with unfunctionalized particles, EGFR is mostly localized in the membrane, the functionalised NP induces its internalization and the EGFR shows a bright intracellular signal in agreement with the expected effect of NPs.

To further support these results, flow cytometry for the cell-surface EGFR was performed for cells treated with: (i) non-functionalised particles, (ii) Ctx alone, and (iii) antibody functionalized particles. For the last category, we employed both Ctx or a combination of Ctx with an antibody against the transferrin receptor (TFR), a receptor known to induce internalisation. Finally, we measure another negative control: NPs with the transferrin antibody alone that are not expected to impact EGFR. The results in the bar plot of Fig. 2C show that the samples having Ctx significantly lower the levels of membrane EGFR as can be appreciated by the lower fluorescence levels, while the bare particles (stacked flow cytometry histogram in Fig. S2A) and particles functionalised with TFR antibodies do not have an effect on the expression of EGFR and show fluorescence intensities comparable to the wildtype negative control. Dual-target particles and single-target particles have a comparable effect on EGFR expression. Flow cytometry histograms of the other particles can be found in Fig. S2B. To explore the role of TFR antibody in EGFR expression when it is on a particle together with Ctx, different ratios of Ctx/TFR were tested (Fig. S2C). This demonstrated that all ratios do have a significant effect on EGFR expression, but the effect is optimal for a 70/30 ratio between the two antibodies. At a lower cetuximab than that, a tail is observed in the histogram, indicating that the particles do not have an effect on the full population of cells. However, this tail is also observed in the 90/10 ratio, indicating that the presence of an optimal concentration of TFR makes the scavenging effect more homogeneous across cells. These measurements show that physicochemical properties such as valency can impact scavenging efficacy and motivate us to explore the design space of the nanoparticles to better understand their structure–activity relations.

### Single-target library

Before exploring the scavenging effect of dual-targeted nanoparticles, the optimal composition of a nanoparticle with only cetuximab was explored. By formulating a NP library of 78 particles, we explored the physicochemical properties of the nanoparticle design space, possibly leading to an optimal particle composition. As discussed before, particles consisted of different materials and sizes and Ctx was conjugated with two different conjugation approaches at multiple different concentrations (Table S1). For visualisation in HCI, fluorescently labelled nanoparticles were used. As the fluorescence of each nanoparticle differs depending on its diameter and material, there is a need for standardization before the functional assays. Furthermore, the extensive washing steps in the synthesis add an additional variability in the fluorescence. To compensate for the differences in fluorescence between particle batches and types and loss during synthesis, a calibration curve for each particle was made, to which the particle fluorescence after functionalisation could be compared and the relative mass of particles could be readdressed (Fig. S3 and Table S2). The calibration curve consisted of the intensities of a dilution series of particles from the original batch, before functionalisation. After functionalisation, the intensity of the particles was compared to this curve to calculate the loss of particles compared to the theoretical concentration. The significant losses of particles that are demonstrated in Table S2 lead to unequal effective concentrations, which can be corrected using the calibration curve. This ensures that any differences observed in cellular uptake reflect effects based on biological differences rather than disparities in fluorescence labelling or particle concentration.

**Fig. 3:**
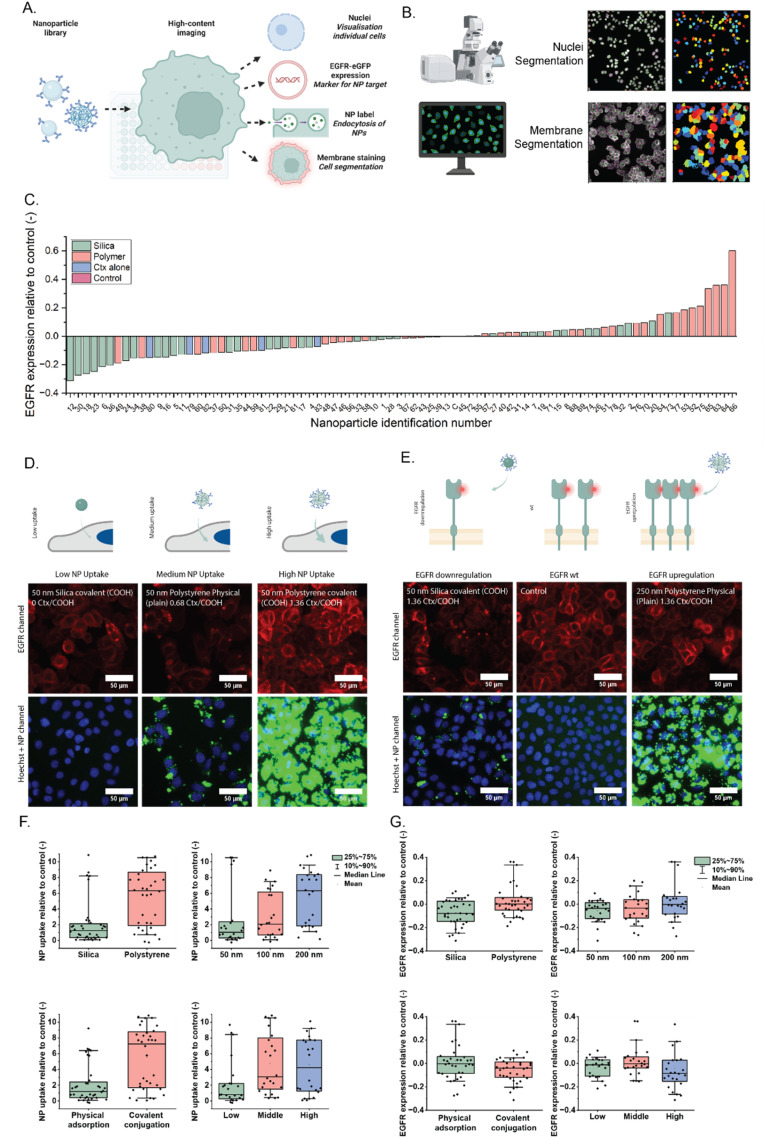
Assessment of a single-target nanoparticle library. (A) Schematic overview of the library formation, treatment of the cells and high-content imaging. MDA-MB-468 cells were transfected to express EGFR-GFP. Cells were stained for nuclei and membrane. (B) Overview of the analysis pipeline. First, the nuclei are segmented to find the individual cells, and then the membrane stain is used for membrane segmentation. Together, these are used to analyze both NP uptake and EGFR expression for each particle. (C) The effect of each NP on the EGFR expression, color coded based on the material of the NP. The bars represent an average value of all cells in 10 ROIs in two wells. Composition of each nanoparticle can be found in Table S1. (D) Representative HCI images of nanoparticle uptake (low, intermediate, and high). (E) Representative HCI images of EGFR expression (WT, downregulation and upregulation). (F) Nanoparticle uptake represented per NP feature. (G) Effect on EGFR expression represented per NP feature.

Following nanoparticle standardization, we designed an HCI cell assay based on 4-color imaging in a high-throughput epifluorescence microscope. The nanoparticle library was introduced to MDA-MB-468 cells stably expressing EGFR-GFP. Particles were added to the cells at the same concentration and after 24-hour incubation, the cells were fixed and stained for their nuclei and membrane using Hoechst and wheat germ agglutinin-Alexa647, respectively. Finally, cells are imaged in an automated way in 96-well plates (Fig. 3A).

The resulting images can then be fed into an image analysis pipeline, based on CellProfiler,^11^ in which the nuclei and membrane staining are used for cell segmentation (Fig. 3B and S4) and the intensity of both the EGFR and nanoparticle signal per cell is quantified.^12^ What can be observed from the EGFR and membrane stain overlays is that in most some cases EGFR is more localized in the inner part of the cell, indicating internalization of the receptor.

The results for EGFR intensity are shown in Fig. 3C for all particles tested. For the nanoparticle uptake, different degrees of internalization were observed, as expected (Fig. 3D and S5), ranging from no uptake to high uptake. We observed distinct nanoparticle-dependent effects on EGFR expression: while some nanoparticles caused a decrease, which indicated receptor scavenging, degradation or downregulation as observed in the initial proof-of-concept, some other samples led to an increase in EGFR levels, suggesting potential receptor signalling upregulation (Fig. 3E). Interestingly, more internalization does not lead *per se* to more scavenging as highlighted in Fig. 3E. Particles with very high uptake may result in less scavenging and *vice versa*. This may indicate that particles with lower tendency to internalize spend more time on the plasma membrane and have higher chance to complex more EGFR receptors. These variations highlight the complexity of nanoparticle–cell interactions and suggest that specific nanoparticle properties influence not only the uptake efficiency^13,14^ but also downstream cellular effects,^15,16^ which are extensively studied for silica particles.^17–19^

By quantifying both the NP uptake efficiency and the EGFR up-/down-regulation, we aim to study structure–activity relations, analysing correlations between different properties of particles and their uptake (Fig. 3F) and scavenging (Fig. 3G). As can be found in Fig. 3G, polystyrene particles mainly stimulate the expression of EGFR, while silica particles mainly scavenge the receptor from the membrane. Similarly, polystyrene particles are more internalized in the cells (Fig. 3F) than silica particles. However, when we look at the nanoparticle surface (plain or COOH) and therewith the conjugation strategy (physisorption *vs.* covalent), physisorbed particles seem to have the highest EGFR upregulation effect, while covalently conjugated particles are internalized more efficiently. To find further correlations, the values of both NP uptake and EGFR expression were plotted against each other (Fig. S6). Apart from the previously discussed differences between effects of different materials, no clear correlation can be found. As the particles are incubated with the cells in media with serum, there is a risk of protein corona formation. The formation of this layer of proteins can have a significant effect on the antibodies bound to the surface of nanoparticles, especially for the physisorption approach.^20^ This effect was assessed by functionalising the particles with fluorescent Ctx and incubating them with serum. This showed that the levels of Ctx were indeed decreasing for physically adsorbed silica nanoparticles, while the other particles had a constant amount of antibodies before and after incubation (Fig. S7), which could explain the lower uptake and minor effect on EGFR expression for the physically adsorbed particles. The remaining signal is also a proxy for the amount of antibody on the surface of the particle. This means that polystyrene particles with covalent attachment have more antibodies bound to their surface compared to all other particles, while the physisorption on the polystyrene particles led to particles with a lower amount of antibodies. The size seems to have a minor influence on EGFR expression, but for the uptake, two populations are found in each diameter. Similarly, 50 nm polystyrene covalent particles have the highest uptake efficacy. The particles that have a high antibody density have a high spread for the EGFR expression, suggesting that more antibody pushes the effect even further (Fig. 3G). Physically adsorbed silica particles all have a low uptake effect. However, these particles still seem to have a high effect on receptor scavenging, with a weak correlation with the original antibody concentration.

### Dual-target library

**Fig. 4:**
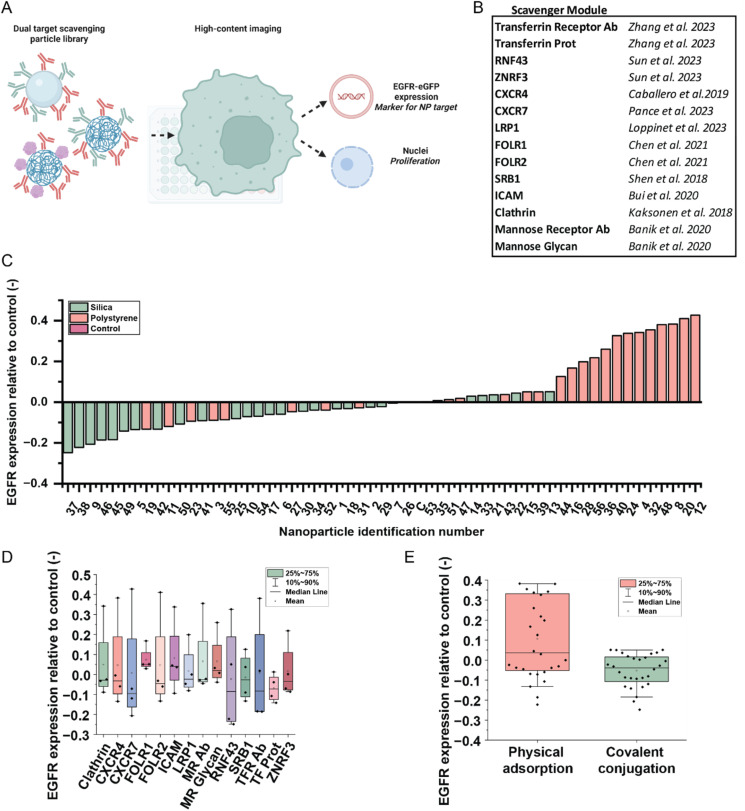
Assessment of a dual-target nanoparticle library. (A) Schematic representation of the approach. First, the NP library with dual targets is formulated. These particles are then added to the cells for HCI. In this case, EGFR expression is assessed. For the hits of both the single-target and dual-target nanoparticle library, a proliferation assay is performed, in which the nuclei are counted. (B) Relevant papers about scavenging modules. These proteins and antibodies are used as the target alongside Ctx. (C) The effect of the NP library on the EGFR expression, color coded by material. The bars represent an average value of all cells in 10 ROIs in two wells. Compositions of the nanoparticles can be found in Table S2. (D) The effect of the NPs on EGFR expression represented per scavenger. (E) The effect of the NPs on EGFR expression represented per conjugation method.

After finding several hits for receptor scavenging in the single-target library and concluding that the material has a large effect on this phenomenon, dual-target particles were introduced. For this library, a similar approach was used. Again, the particles were incubated with the cells and their uptake and effect on EGFR expression were assessed using HCI and the CellProfiler pipeline (Fig. 4A). Fourteen proteins and antibodies were selected for the scavenging module, all previously shown to have a scavenging potential in the literature before (Fig. 4B) as they are known to be involved in the endocytosis pathway, or they were shown to be potential PROTACs, LYTACs, or other protein degradation platforms.^21–31^ All scavengers were commercially available, except for the mannose glycan E6, which was synthesised in our lab.^31^ Together with Ctx, each of these scavengers was again immobilized on particles with different materials, using the two conjugation strategies shown before. This time, the diameter of the particles was kept constant at 200 nm and the Ctx concentration was kept constant at 0.68 Ctx/COOH, based on the results of the previous section. This resulted in a library of 56 particles that we again analysed for their uptake efficacy and scavenging potential (Table S3).

As shown in Fig. 4C, each particle had a different effect on the EGFR expression. Again, a part of the particles induced EGFR downregulation and thus demonstrated a desired scavenging effect, while other particles upregulated the expression of EGFR. More specifically, a large majority of the scavenger particles are made up of a silica core, while the polystyrene particles are mostly upregulating the expression. When grouping the particles based on their scavenger (Fig. 4D), it becomes clear that apart from the FOLR1 and TFR antibody particles, the effect is very heterogeneous, suggesting that the type of scavenging module is not the only determinant of the eventual result. When grouping them based on the conjugation method (Fig. 4E), it becomes clear that the upregulating particles are often the physically adsorbed particles, functionalized with physisorption, while EDC-conjugated particles either have no effect or have a scavenging effect. Compared to the single-target library, this effect is more pronounced. Among the scavenger modules, the transferrin protein seems to enable at the same time the most effective EGFR scavenging and the least heterogeneous results, indicating that the natural ligand for an internalizing receptor seems a robust and effective strategy for NP-based scavengers. It remains unclear if the particles have a direct effect on the upregulation of receptor expression, or through a successful scavenging effect over the course of the incubation which resulted in overproduction of the receptor, opposed to the desired effect of EGFR downregulation and possible inhibition of cell proliferation.^8,32–35^ This latter effect is demonstrated when plotting the EGFR expression over the NP uptake. Most of the polystyrene particles show a high uptake, but they display upregulation of the receptor. However, all EDC-conjugated particles show a synergy between uptake and EGFR scavenging (Fig. S8 and S9). To assess the overall effect of the particles on the cell viability, a proliferation assay was performed (Fig. S10). Particles from the single-target library show a stable nuclei count compared to the control, while dual-target particles lower the proliferation of the cells, indicated by the lower nuclei count. This underscores the effect of dual-target nanoparticles on cell fate.

## Discussion

This study presents a high-content screening approach for a large NP library designed to modulate EGFR expression *via* targeted scavenging, leveraging antibody-functionalized nanoparticles as a modular platform for membrane receptor depletion. While previous work has demonstrated targeted receptor degradation using antibodies alone,^23,36^ our approach leverages nanoparticle design as a modular and tuneable platform for influencing receptor fate at the cell surface. By systematically varying the NP material, diameter, scavenger module type and cetuximab concentration, and assessing their effect in a high-throughput fashion, we identified several design-dependent outcomes in EGFR expression, cellular uptake and proliferative response.

Because the screening platform relies on fluorescence-based readouts for nanoparticle uptake and effect on EGFR, accurate interpretation requires that measurements are not biased by differences in nanoparticle concentration and fluorescence. Therefore, before adding the particles to the cells, a standardization step was performed to account for these potential sources of variation. Calibration curves of fluorescence *versus* concentration showed that fluorescence intensity was independent of size, but silica particles were significantly less fluorescent than polystyrene particles. In addition, loss of particles during synthesis was around 55% for the silica nanoparticles, while the polystyrene particles on average had a loss of 80%, leading to unequal effective concentrations if they remained uncorrected. These results underscore the importance of pre-experimental normalization when comparing effects of nanoparticles in a high-throughput setting.

A key observation was the bimodal effect of cetuximab-functionalized single-target NPs on EGFR expression levels. Depending on the particle material, conjugation method and to some degree on antibody concentration, EGFR expression was either enhanced or significantly reduced. This dual behaviour suggests that NP design plays a critical role in modulating receptor trafficking.

The dual-targeting NPs, functionalized with both Ctx and a scavenger module, exhibited similar trends in EGFR scavenging to the single-target versions. The material of the particle was the determining factor in the effectiveness of receptor scavenging, outweighing the influence of scavenger type.^32–34^ Within the silica particle subset, anti-RNF43, TFR antibodies and the ligand and anti-CXCR7 stood out as strong candidates, producing receptor depletion reminiscent of LYTAC mechanisms. These results suggest that an ideal NP for further development would combine silica as the core material with one of these scavenger modules, offering a starting point for further optimization of the library. In addition to material and scavenger choice, particle shape is another parameter likely to influence internalization and scavenging efficiency and should be systematically explored in these future libraries.

Despite the applicability of the screening platform, several limitations should be considered. While high-content imaging enables efficient and multiplexed phenotyping of many samples at once and at the single-cell level, it lacks the resolution to capture details at the single-molecule level. Techniques such as single-particle tracking or super-resolution microscopy could provide a deeper insight into receptor engagement and nanoparticle architecture. For example, the number of antibody Fab domains exposed on the surface of the particle could be counted using super-resolution microscopy.^37^ However, these approaches are inherently low-throughput, making them best suited for detailed follow-up on selected hits rather than for screening entire libraries. Combining high-throughput screening with targeted single-particle analyses would therefore provide a multilevel framework for linking the nanoparticle structure to function. Nonetheless, one needs to consider that batch-to-batch variability (particle and antibody) and researcher-to-researcher variability, as demonstrated by our previous work concerning nanoparticle generation difference between researchers, have a significant effect on the composition of the nanoparticles and thus also on the effect in cells.^37^

This also leads to the second limitation; this batch-to-batch variation can significantly influence the biological outcomes, potentially hampering reproducibility. All experiments presented here are based on a single batch of particles to avoid variability, so further studies should systematically assess its impact. Next, only a single cell model was used. Further studies with other cell lines with the same library would indicate if our observations were based on the nature of the material or the cell in question. Furthermore, we only targeted EGFR as it is an important cancer therapeutic target, but this protein has its own pattern of regulation. Particles that scavenge this receptor might or might not be able to scavenge other proteins to the same extent. Therefore, further studies that assess multiple receptors would provide more insights into material properties related to the effect.

Taken together, our approach offers a quantitative framework to explore nanoparticle-mediated receptor scavenging, using high-content imaging. This mode of imaging allows for evaluation of complex bio–nano interactions at the single-cell level. The programmability of the approach allows for minor changes in the pipeline for the question at hand. We envision that this will contribute to rationalised exploration of the nanoparticle design space, leading to more understanding of design requirements as well as a decrease in the time spent on optimisation. This will speed-up the concept-to-therapy pipeline for nanoparticles significantly.

## Experimental

### Reagents

Sicastar-GreenF plain, Sicastar-RedF plain (200 nm, 100 nm, and 50 nm), Sicastar-RedF COOH (200 nm, 100 nm, and 50 nm), Micromer-RedF plain (250 nm, 100 nm, and 50 nm) and Micromer-RedF COOH (250 nm, 100 nm, and 50 nm) were purchased from Micromod Partikeltechnologie GmbH. Paraformaldehyde (PFA) 16% solution (043368-9M) was purchased from Thermo Fisher Scientific. 1-Ethyl-3-(3-dimethylaminopropyl)-carbodiimide (EDC), 2-morpholin-4-ylethanesulfonic acid (MES) (50 mM, pH 5.5) and phosphate-buffered saline (PBS) were purchased from Sigma Aldrich. Hoechst 33342 20 mM (Invitrogen 62249) and wheat germ agglutinin (WGA) Alexa Fluor 637 conjugate (Invitrogen W32466) were purchased from Fisher Scientific.

### Antibodies and targeting ligands

Cetuximab was provided by collaborators (Anna Świetlikowska), and in-house conjugated with Alexa Fluor 680. Cetuximab-Alexa647 (FAB9577R) was purchased from R&D, and used for FACS experiments. Anti-EGFR antibody (targeting cytoplasmic tail, clone 8G6.2) was purchased from Merck, conjugated with Alexa647, and used for confocal experiments. Transferrin receptor (OKT9, 14-0719-82), ICAM (PA1-29968), Folate Receptor (MA5-23917), CXCR4 (35-8800), and Clathrin Heavy Chain (MA1-065) antibodies were purchased from Invitrogen. Folate Receptor 2 (AB288431), SRB1 (AB106572) and Mannose Receptor (AB64693) antibodies were purchased from Abcam. LRP1 (LS-C775116) antibody was purchased from LSbio. hRNF43 (MAB7964) and hCXCR7 (MAB42273) antibodies were purchased from R&D. ZRNF3 (NBP3-03924) antibody was purchased from Novus. The mannose receptor glycan functionalisation consisted of the E6 mannose previously synthesised batched fro Riera *et al.* (2021).^31^

### Chemical conjugation and absorption of cetuximab to silica and polymer NPs

Cetuximab was attached to Sicastar-redF COOH and Micromer-redF COOH *via* EDC conjugation or physisorption. For EDC conjugation, the NPs were spun down by centrifugation for 10 min at 16 000 × *g* in order to remove the storage solution and then washed in MES buffer. Later, the NPs were resuspended in MES buffer with EDC (1 mg ml^−1^) and incubated for 15 min at RT at 450 rpm in a thermomixer. After incubation, the NPs were sonicated for 1 min in a sonication bath. Different concentrations of cetuximab were added based on the antibody molecule/COOH group (0.17/0.34/0.67/1.02/1.34). After addition of cetuximab, the NPs were incubated in a thermomixer at RT at 450 rpm for 2 hours. Afterwards, the NPs were washed with MES buffer three times at 16 000 × *g* for 15 min. At the end, the NPs were resuspended at a final concentration of 1 mg ml^−1^ in PBS and stored in a fridge. Plain NPs were similarly attached with cetuximab through absorption, using the above protocol without the presence of EDC.

### Measuring actual NP sample concentration and confirming antibody labelling using fluorescence

Different concentrations of unlabeled NPs were measured using a Varioskan LUX microplate reader, to assess a baseline of NP concentration per fluorescent expression. After NP antibody labelling, the NP samples were measured using a Varioskan LUX microplate reader and the fluorescence (expressed by the NPs) was compared to the baseline to assess actual NP concentration after labelling. The actual concentration was used in subsequent experiments. Cetuximab Alexa 680 was physically absorbed to Sicastar-RedF plain (100 nm) according to the above protocol. Afterwards half of the NP solution was incubated in 10% fetal bovine serum (FBS) for 15 min at RT and 450 rpm in a thermomixer, in order to create a protein corona. After incubation, the samples were washed one time in MES by centrifugation at 16 000 × *g* for 15 min. Thereafter, the samples were analysed for fluorescence using a Varioskan LUX microplate reader to assess antibody labelling and whether protein corona formation interferes with antibody labelling.

### Cell culture and transfection

Immortalised breast cancer cell line MDA-MB-468, which highly expresses EGFR, was purchased from ATCC and cultured in Gibco Dulbecco's Modified Eagle Medium (DMEM), with 10% FBS and 1% penicillin/streptomycin under standard cell culture conditions (37 °C and 5% CO_2_). Cells were passaged every 2 to 3 days when around 80% confluence was reached. Passaging was performed by washing the cells with Dulbecco's PBS 1× and detached using Gibco's non-enzymatic cell dissociation buffer. For FACS experiments, cells were plated in a 24-well plate, 5 × 10^4^ per well, and every condition in duplicate. For confocal imaging or high throughput screening (HTS) experiments, we seeded the cells in black 96-well plates with an Ibidi-Treat optical bottom (89626) from Ibidi with a density of 1 × 10^4^ cells per well. The MDA-MB-468 cells were transfected with EGFR-eGFP construct purchased from AddGene (32751), using lipofectamine 3000 from Thermo Fisher Scientific and the cell appropriate protocol as suggested by the company. After transfection, cells were sorted using FACS for fluorescent-expressing cells and kept on G418 antibiotic selection during cell passaging (not during the experimental phase).

### Confocal imaging

For the confocal imaging, MDA-MB-468 cells were plated in an optical 96-well plate and incubated overnight at 37 °C. The next day, Sicastar-GreenF plain (100 nm) with and without antibody labelling were added, 10 µg per well, for 24 hours at 37 °C. After this time, cells were incubated with Hoechst for 30 minutes at 37 °C, and subsequently washed with DPBS and fixed using 2% PFA for 10 minutes at RT, and after that washed 2× with DPBS. The fixed cells were then incubated with TritonX100/PBS 0.04% for 5 minutes at RT, and washed 3 times with PBS before incubation with anti-EGFR Alexa647 (intracellular) for 1 hour at RT. Cells were washed 3× with PBS and imaged using a Nikon Eclipse Ti-E microscope. The microscope was operated using Nikon Instrument Software (NIS) elements. Imaging consisted of taking 5 pictures per well, Hoechst nuclei 405 nm, NPs 568 nm, and EGFR 647 nm. Images were processed and visualized with ImageJ FIJI.

### FACS analysis

MDA-MB-468 cells were plated in a 24-well plate and incubated ON at 37 °C. Cells were incubated with Sicastar-GreenF plain (100 nm) with and without antibody labelling, 50 µg per well, in duplicate, for 24 hours at 37 °C. After incubation, cells were washed with DPBS and detached from the plate using a non-enzymatic dissociation buffer. Duplicates were pooled and 4% PFA/PBS solution was added in a volume to create a 2% PFA/PBS working concentration. Cells were fixed for 10 minutes at RT. Cells were centrifuged at 250 × *g* to remove PFA and 1 mL PBS was added. Cetuximab-Alexa647 was added to the samples for 30 minutes at RT, centrifuged to remove the antibody, 1 mL PBS was added and transferred to an FACS tube and then kept on ice while moving towards FACS Canto I for analysis. FlowJo was used for analysis and histogram visualization. Cells were gated for singlet cells and Alexa647 was measured as a representation for EGFR expression.

### High throughput screening

For the HTS, we used MDA-MB-468 cells transfected with EGFR-eGFP and seeded in optical 96-well plates overnight at 37 °C. The following day, the medium was renewed, Sicastar-RedF particles were added to the wells, 5 µg per well, in duplicate and incubated for 24 hours at 37 °C. After the incubation, the medium was renewed, with an addition of Hoechst (0.4 µM) and WGA-637 (5 µg mL^−1^), for 30 minutes at 37 °C. Afterwards, the medium was removed, cells were washed with DPBS, fixed with 2% PFA for 10 minutes at RT and subsequently washed 2× with PBS and kept in PBS at 4 °C. Imaging took place in the same week. For the screening, widefield microscopy images were acquired using a Nikon Eclipse Ti_2_ microscope adapted for high content. The microscope consists of an automated piezo stage and focus system, a 25-mm prim∑ 95B sMOS camera from Teledyne Photometrics (Arizona, USA), a Spectra X light engine from Lumencor (Oregon, USA) and a live-cell stage incubator from Okolab (Pozzuoli, Italy). The microscope was operated using Nikon Instrument Software (NIS) elements. Imaging consisted of taking 5 fields of view (FOVs) per well, Hoechst nuclei 405 nm, EGFR-eGFP 488 nm, NPs 568 nm, and WGA 637 nm. A pipeline for automated imaging was set using Nikon's JOBS macro. Analysis of EGFR expression was performed using CellProfiler, according to Ortiz Perez *et al.*^12^ Afterwards, averaged fluorescence intensities of the NP and EGFR channels were normalized against the respective controls.

## Author contributions

Conceptualization was done by J. O., M. M. E. T. and L. A. Methodology was formulated by J. O., M. M. E. T., A. O. P. and L. A. All experiments were performed by J. O., J. G. D. D. and V. G. Data analysis was performed by J. O. and A. O. P. The original draft was written by M. M. E. T. and L. A. in consultation with J. O., J. D., A. O. P. and V. G.

## Conflicts of interest

The authors declare no competing financial interest.

## Supplementary Material

NA-008-D5NA00811E-s001

## Data Availability

Data will be available on the GitHub of the N4Nlab (github.com/n4nlab/HT_scavenging). This repository contains the final Origin files with the data of the high-content imaging and the CellProfiler pipeline. Supplementary information is available. See DOI: https://doi.org/10.1039/d5na00811e.
