# Supplementary material for: A high-throughput approach to optimize and understand nanoparticle protein degraders
Source: Nanoscale Adv. 2025 Nov 17;8(2):479–89. doi: 10.1039/d5na00811e (PMC12666540; doi:10.1039/d5na00811e)
Supplement: NA-008-D5NA00811E-s001 [file NA-008-D5NA00811E-s001.pdf]

Supplementary Information (SI) for Nanoscale Advances.
This journal is © The Royal Society of Chemistry 2025

Supplementary information

Optimizing nanoparticle protein scavengers through high-throughput screening

Joppe Oldenburg[a], Marrit M.E. Tholen[a], Janne G. D. Donkers[a], Ana Ortiz-Perez[a], Valentina Girola[a], Lorenzo Albertazzi[a]*

[a] Department of Biomedical Engineering, Institute for Complex Molecular Systems (ICMS), Eindhoven University of Technology, Eindhoven 5612AZ, The Netherlands.

* l.albertazzi@tue.nl

Supplementary Tables

Table S. 1: Single-target library formulation and description for the cellular uptake tests in Fig. 3.

Number	Formulation name	Material	Diameter (nm)	Amount of Ctx (Ctx/COOH)	Conjugation method
1	S_50_plain_1	Silica	50	0	Physisorption
2	S_50_plain_2	Silica	50	0.17	Physisorption
3	S_50_plain_3	Silica	50	0.34	Physisorption
4	S_50_plain_4	Silica	50	0.68	Physisorption
5	S_50_plain_5	Silica	50	1.02	Physisorption
6	S_50_plain_6	Silica	50	1.36	Physisorption
7	S_50_COOH_1	Silica	50	0	Covalent
8	S_50_COOH_2	Silica	50	0.17	Covalent
9	S_50_COOH_3	Silica	50	0.34	Covalent
10	S_50_COOH_4	Silica	50	0.68	Covalent
11	S_50_COOH_5	Silica	50	1.02	Covalent
12	S_50_COOH_6	Silica	50	1.36	Covalent
13	S_100_plain_1	Silica	100	0	Physisorption
14	S_100_plain_2	Silica	100	0.17	Physisorption
15	S_100_plain_3	Silica	100	0.34	Physisorption
16	S_100_plain_4	Silica	100	0.68	Physisorption
17	S_100_plain_5	Silica	100	1.02	Physisorption
18	S_100_plain_6	Silica	100	1.36	Physisorption
19	S_100_COOH_1	Silica	100	0	Covalent
20	S_100_COOH_2	Silica	100	0.17	Covalent
21	S_100_COOH_3	Silica	100	0.34	Covalent
22	S_100_COOH_4	Silica	100	0.68	Covalent
23	S_100_COOH_5	Silica	100	1.02	Covalent
24	S_100_COOH_6	Silica	100	1.36	Covalent
25	S_200_plain_1	Silica	200	0	Physisorption
26	S_200_plain_2	Silica	200	0.17	Physisorption
27	S_200_plain_3	Silica	200	0.34	Physisorption
28	S_200_plain_4	Silica	200	0.68	Physisorption
29	S_200_plain_5	Silica	200	1.02	Physisorption
30	S_200_plain_6	Silica	200	1.36	Physisorption
31	S_200_COOH_1	Silica	200	0	Covalent
32	S_200_COOH_2	Silica	200	0.17	Covalent
33	S_200_COOH_3	Silica	200	0.34	Covalent
34	S_200_COOH_4	Silica	200	0.68	Covalent
35	S_200_COOH_5	Silica	200	1.02	Covalent
36	S_200_COOH_6	Silica	200	1.36	Covalent
37	P_50_plain_1	Polystyrene	50	0	Physisorption
38	P_50_plain_2	Polystyrene	50	0.17	Physisorption
39	P_50_plain_3	Polystyrene	50	0.34	Physisorption
40	P_50_plain_4	Polystyrene	50	0.68	Physisorption
41	P_50_plain_5	Polystyrene	50	1.02	Physisorption
42	P_50_plain_6	Polystyrene	50	1.36	Physisorption
43	P_50_COOH_1	Polystyrene	50	0	Covalent
44	P_50_COOH_2	Polystyrene	50	0.17	Covalent
45	P_50_COOH_3	Polystyrene	50	0.34	Covalent
46	P_50_COOH_4	Polystyrene	50	0.68	Covalent
47	P_50_COOH_5	Polystyrene	50	1.02	Covalent
48	P_50_COOH_6	Polystyrene	50	1.36	Covalent
49	P_100_plain_1	Polystyrene	100	0	Physisorption

50	P_100_plain_2	Polystyrene	100	0.17	Physisorption
51	P_100_plain_3	Polystyrene	100	0.34	Physisorption
52	P_100_plain_4	Polystyrene	100	0.68	Physisorption
53	P_100_plain_5	Polystyrene	100	1.02	Physisorption
54	P_100_plain_6	Polystyrene	100	1.36	Physisorption
55	P_100_COOH_1	Polystyrene	100	0	Covalent
56	P_100_COOH_2	Polystyrene	100	0.17	Covalent
57	P_100_COOH_3	Polystyrene	100	0.34	Covalent
58	P_100_COOH_4	Polystyrene	100	0.68	Covalent
59	P_100_COOH_5	Polystyrene	100	1.02	Covalent
60	P_100_COOH_6	Polystyrene	100	1.36	Covalent
61	P_250_plain_1	Polystyrene	250	0	Physisorption
62	P_250_plain_2	Polystyrene	250	0.17	Physisorption
63	P_250_plain_3	Polystyrene	250	0.34	Physisorption
64	P_250_plain_4	Polystyrene	250	0.68	Physisorption
65	P_250_plain_5	Polystyrene	250	1.02	Physisorption
66	P_250_plain_6	Polystyrene	250	1.36	Physisorption
67	P_250_COOH_1	Polystyrene	250	0	Covalent
68	P_250_COOH_2	Polystyrene	250	0.17	Covalent
69	P_250_COOH_3	Polystyrene	250	0.34	Covalent
70	P_250_COOH_4	Polystyrene	250	0.68	Covalent
71	P_250_COOH_5	Polystyrene	250	1.02	Covalent
72	P_250_COOH_6	Polystyrene	250	1.36	Covalent
73	S_50_plain_4	Silica	50	0.68	Physisorption
74	S_50_COOH_4	Silica	50	0.68	Covalent
75	P_50_Plain_4	Polystyrene	50	0.68	Physisorption
76	P_50_COOH_4	Polystyrene	50	0.68	Covalent
77	P_100_plain_4	Polystyrene	100	0.68	Physisorption
78	P_100_COOH_4	Polystyrene	100	0.68	Covalent
79	Cetuximab_1	-	-	0.17	-
80	Cetuximab_2	-	-	0.34	-
81	Cetuximab_3	-	-	0.68	-
82	Cetuximab_4	-	-	1.02	-
83	Cetuximab_5	-	-	1.36	-
C	Control	-	-	-	-

Table S. 2: Average percentage of NPs left when expecting 1 mg/mL. * It should be noted that the samples are severely aggregated, which may affect the reliability of the results.

**Silica**	**Percentage left**	**Polystyrene**	**Percentage left**
Plain 50 nm	49.9%	Plain 50 nm	0.4%
Plain 100 nm	52.8%	Plain 100 nm	8.8%
Plain 200 nm	66.8%	Plain 250 nm	27.8%
COOH 50 nm	21.1%	COOH 50 nm	4.9%
COOH 100 nm	32.3%*	COOH 100 nm	23.1%
COOH 200 nm	42.7%	COOH 250 nm	58.2%

Table S. 3: Dual-target library formulation and description for the cellular uptake tests in Fig. 4. Diameter: 200 nm for the silica particles, 250 nm for the polystyrene particles. Ctx concentration: 0.68 Ctx/COOH.

**Number**	**Formulation name**	**Material**	**Conjugation method**	**Scavenging protein**
1	S_COOH_Clath	Silica	COOH	Clathrin
2	S_Plain_Clath	Silica	Plain	Clathrin
3	P_COOH_Clath	Polystyrene	COOH	Clathrin

4	P_Plain_Clath	Polystyrene	Plain	Clathrin
5	S_COOH_CXCR4	Silica	COOH	CXCR4
6	S_Plain_CXCR4	Silica	Plain	CXCR4
7	P_COOH_CXCR4	Polystyrene	COOH	CXCR4
8	P_Plain_CXCR4	Polystyrene	Plain	CXCR4
9	S_COOH_CXCR7	Silica	COOH	CXCR7
10	S_Plain_CXCR7	Silica	Plain	CXCR7
11	P_COOH_CXCR7	Polystyrene	COOH	CXCR7
12	P_Plain_CXCR7	Polystyrene	Plain	CXCR7
13	S_COOH_FOLR1	Silica	COOH	FOLR1
14	S_Plain_FOLR1	Silica	Plain	FOLR1
15	P_COOH_FOLR1	Polystyrene	COOH	FOLR1
16	P_Plain_FOLR1	Polystyrene	Plain	FOLR1
17	S_COOH_FOLR2	Silica	COOH	FOLR2
18	S_Plain_FOLR2	Silica	Plain	FOLR2
19	P_COOH_FOLR2	Polystyrene	COOH	FOLR2
20	P_Plain_FOLR2	Polystyrene	Plain	FOLR2
21	S_COOH_ICAM	Silica	COOH	ICAM
22	S_Plain_ICAM	Silica	Plain	ICAM
23	P_COOH_ICAM	Polystyrene	COOH	ICAM
24	P_Plain_ICAM	Polystyrene	Plain	ICAM
25	S_COOH_LRP1	Silica	COOH	LRP1
26	S_Plain_LRP1	Silica	Plain	LRP1
27	P_COOH_LRP1	Polystyrene	COOH	LRP1
28	P_Plain_LRP1	Polystyrene	Plain	LRP1
29	S_COOH_MRAb	Silica	COOH	MR Ab
30	S_Plain_MRAb	Silica	Plain	MR Ab
31	P_COOH_MRAb	Polystyrene	COOH	MR Ab
32	P_Plain_MRAb	Polystyrene	Plain	MR Ab
33	S_COOH_MRGlycan	Silica	COOH	MR Protein
34	S_Plain_MRGlycan	Silica	Plain	MR Protein
35	P_COOH_MRGlycan	Polystyrene	COOH	MR Protein
36	P_Plain_MRGlycan	Polystyrene	Plain	MR Protein
37	S_COOH_RNF43	Silica	COOH	RNF43
38	S_Plain_RNF43	Silica	Plain	RNF43
39	P_COOH_RNF43	Polystyrene	COOH	RNF43
40	P_Plain_RNF43	Polystyrene	Plain	RNF43
41	S_COOH_SRB1	Silica	COOH	SRB1
42	S_Plain_SRB1	Silica	Plain	SRB1
43	P_COOH_SRB1	Polystyrene	COOH	SRB1
44	P_Plain_SRB1	Polystyrene	Plain	SRB1
45	S_COOH_TFRAb	Silica	COOH	TFR Ab
46	S_Plain_TFRAb	Silica	Plain	TFR Ab
47	P_COOH_TFRAb	Polystyrene	COOH	TFR Ab
48	P_Plain_TFRAb	Polystyrene	Plain	TFR Ab
49	S_COOH_TFRProt	Silica	COOH	TFR Protein
50	S_Plain_TFRProt	Silica	Plain	TFR Protein
51	P_COOH_TFRProt	Polystyrene	COOH	TFR Protein
52	P_Plain_TFRProt	Polystyrene	Plain	TFR Protein
53	S_COOH_ZNRF3	Silica	COOH	ZNRF3
54	S_Plain_ZNRF3	Silica	Plain	ZNRF3
55	P_COOH_ZNRF3	Polystyrene	COOH	ZNRF3
56	P_Plain_ZNRF3	Polystyrene	Plain	ZNRF3
C	Control	-	-	-

Supplementary Figures

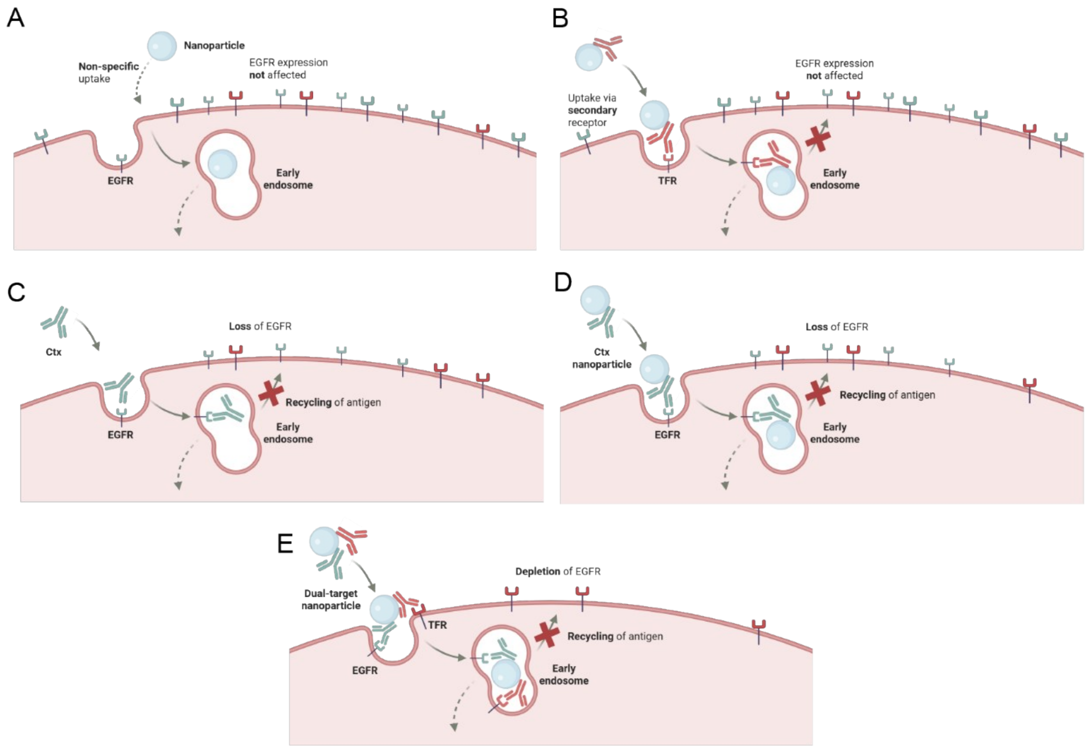

Figure S 1: Proposed mechnisms of uptake. A) Nanoparticles that are not functionalised will be internalized via non-specific uptake and will not have an effect on EGFR expression. B) Nanoparticles functionalized with a scavenging antibody will get internalized, taking their receptor with them, but have no effect on EGFR. C) Cetuximab will be internalized and will have a downregulating effect on EGFR. D) Nanoparticles with Ctx will have the same effect as Ctx alone. E) Dual-target NPs will bind to both receptors and result in significant reduction in EGFR expression.

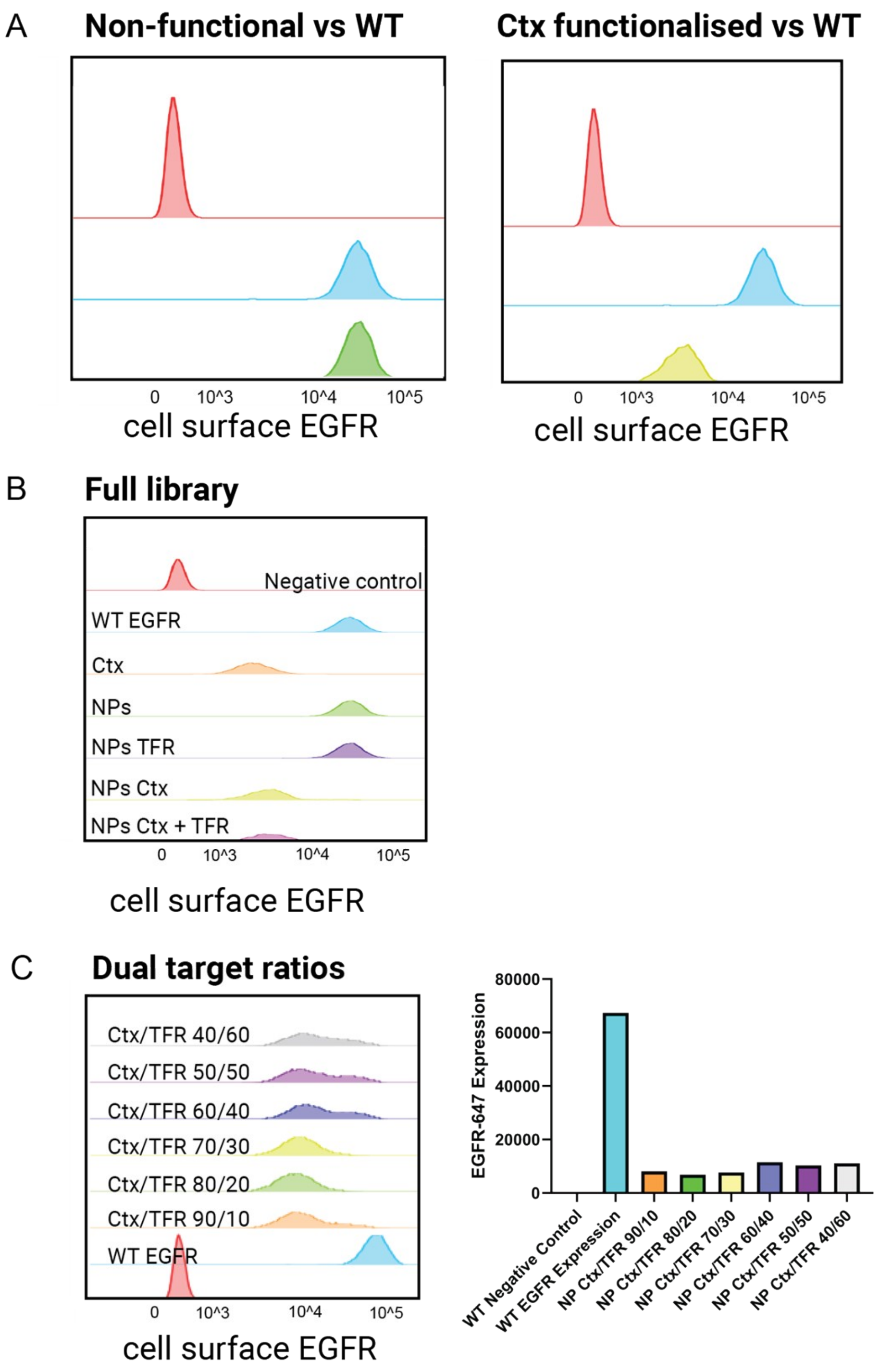

Figure S 2: Flow cytometry of nanopartilces. A) Stacked flow cytometry histograms of the data presented in Fig. 2C i) and ii). B) Flow cytometry histograms of all data presented in Fig. 2C. Material of the nanoparticles is silica. iii) C) FCS results of double functionalised silica NPs with Ctx and TFR in different ratios.

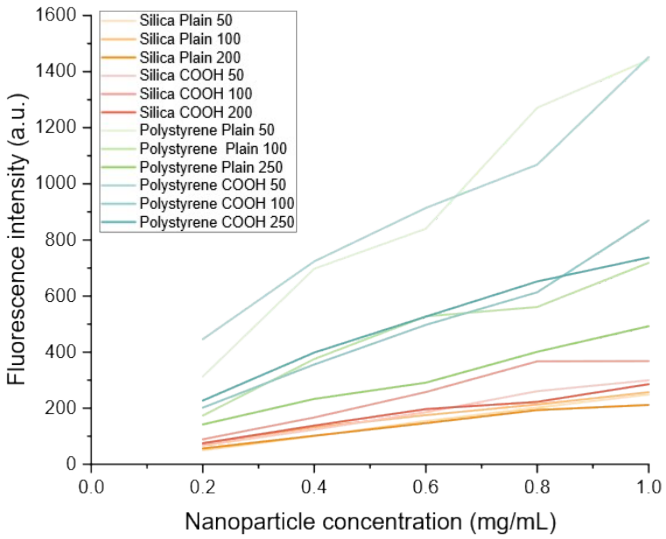

Figure S 3: Calibration curves for the nanoparticle types.

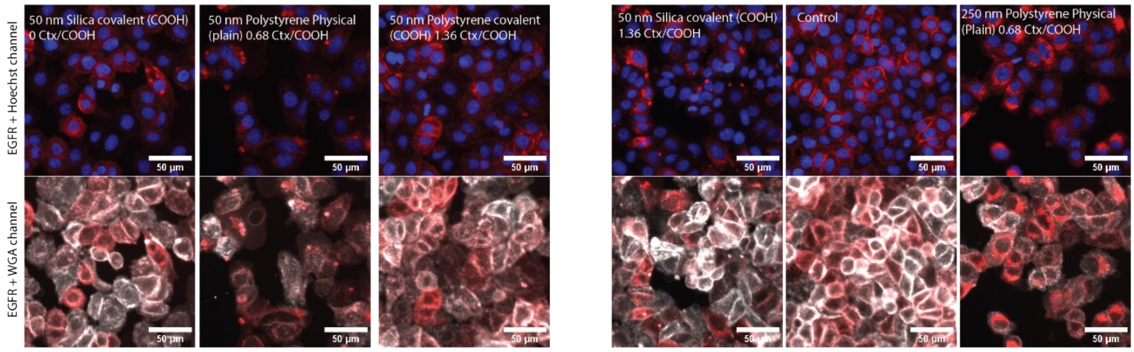

Figure S 4: Figures accompanying images shown in Fig. 3. Top row: EGFR and Hoechst channel overlay, bottom row: EGFR and WGA channel overlay.

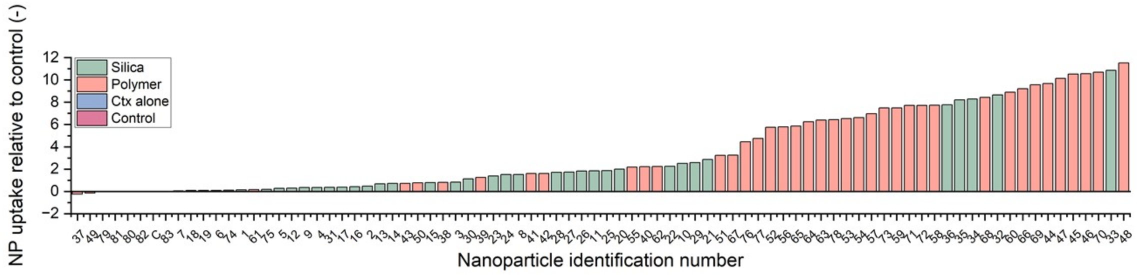

Figure S 5: Single-target nanoparticle uptake compared to control, color coded based on nanoparticle core material.

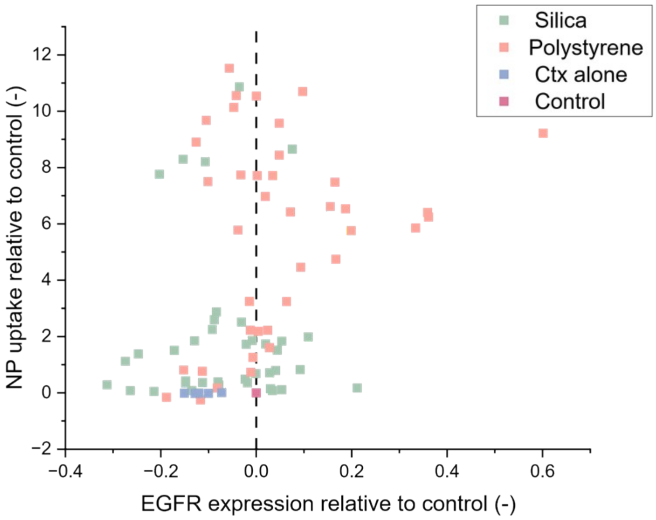

Figure S 6: Scatterplot of the EGFR expression over NP uptake after treatment with different NPs.

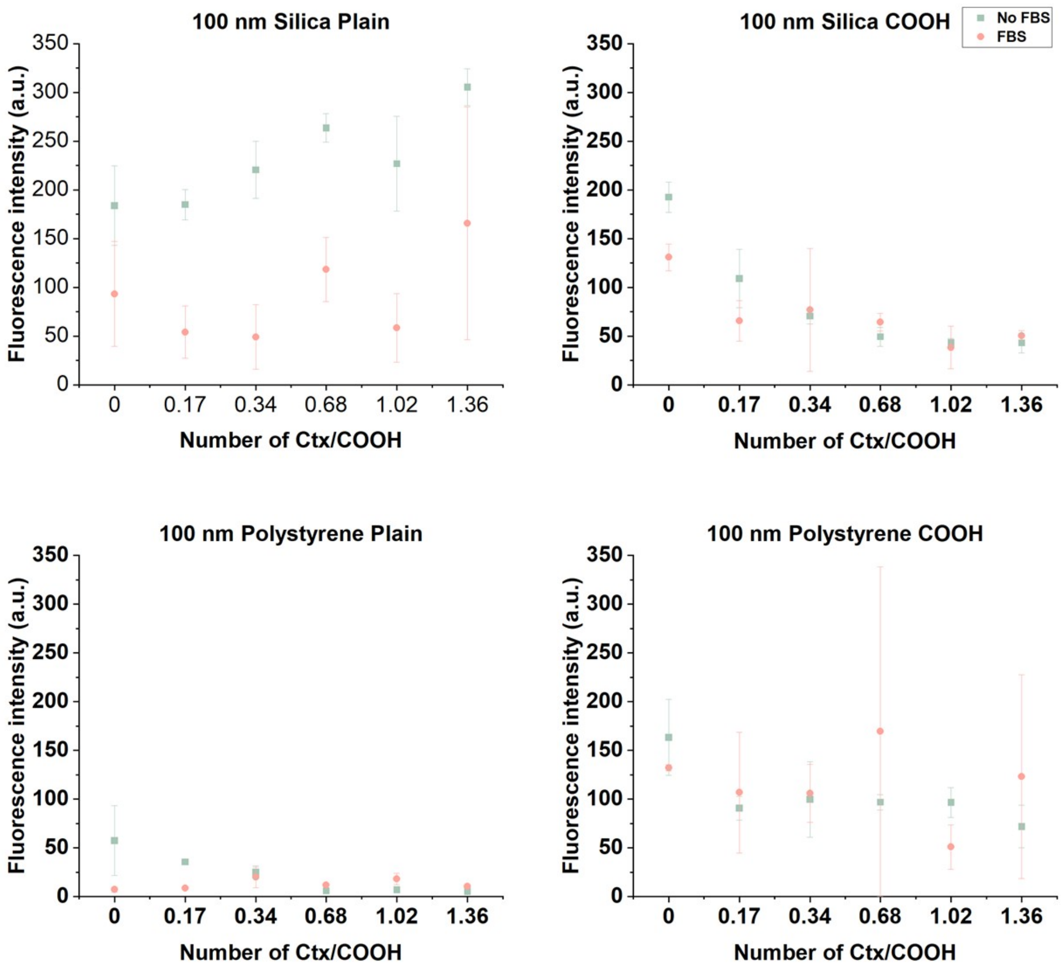

Figure S 7: **Fluorescence intensities of nanoparticles before and after incubation with serum.** Particles were functionalised with fluorescent Ctx, batch was splitted in two and one half was incubated with serum and

washed. Difference between 100 nm Silica Plain are significantly different (p<0.001), others are not significantly different. This indicates a loss in Abs for the first case, no loss in the other cases.

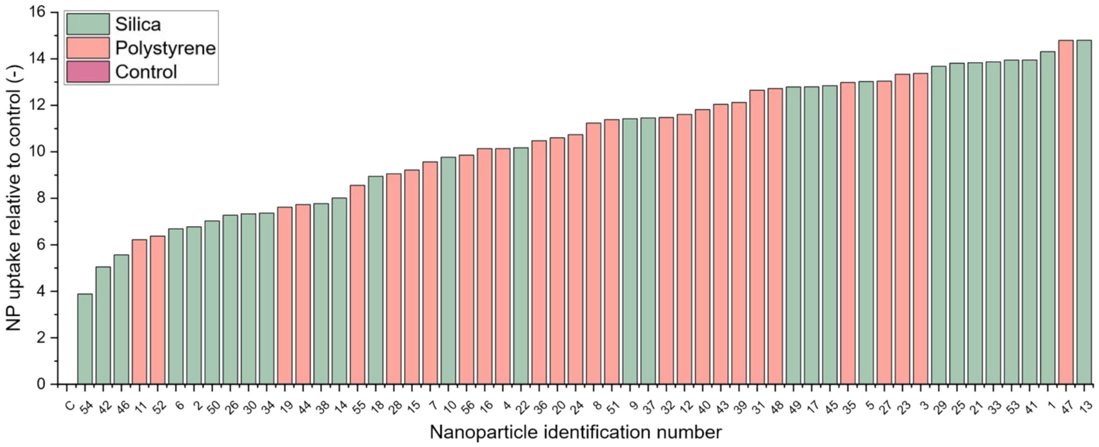

Figure S 8: Dual-target nanoparticle uptake compared to control, color coded based on nanoparticle core material.

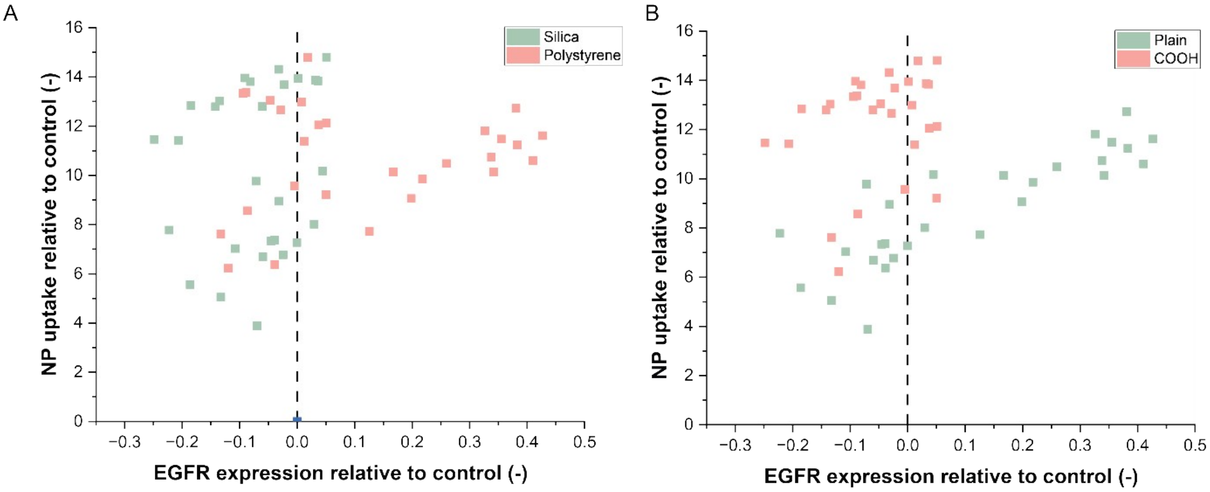

Figure S 9: Scatterplots of EGFR expression over NP uptake for the dual targeted particles. A) Colour coded by material, B) colour coded by conjugation strategy. Dotted line indicates value observed for control.

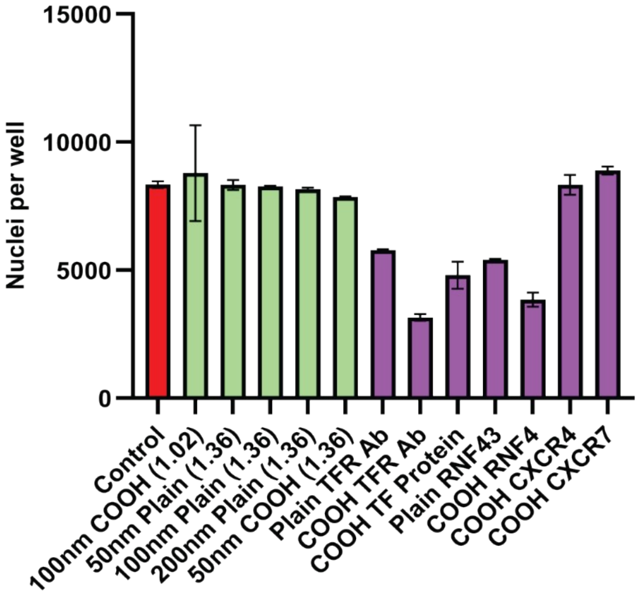

Figure S 10: Proliferation test of cells after treatment with different nanoparticles. Red indicates control, green bars represent nanoparticles from the single-target library and purple bars represent particles from the dual-target library.
